# Diffusion MRI quality control and functional diffusion map results in ACRIN 6677/RTOG 0625: A multicenter, randomized, phase II trial of bevacizumab and chemotherapy in recurrent glioblastoma

**DOI:** 10.3892/ijo.2015.2891

**Published:** 2015-02-11

**Authors:** BENJAMIN M. ELLINGSON, EUNHEE KIM, DAVIS C. WOODWORTH, HELGA MARQUES, JERROLD L. BOXERMAN, YAIR SAFRIEL, ROBERT C. McKINSTRY, FELIX BOKSTEIN, RAJAN JAIN, T. LINDA CHI, A. GREGORY SORENSEN, MARK R. GILBERT, DANIEL P. BARBORIAK

**Affiliations:** 1 UCLA Neuro-Oncology Program, David Geffen School of Medicine, University of California Los Angeles, Los Angeles, CA; 2 Department of Radiological Sciences, David Geffen School of Medicine, University of California Los Angeles, Los Angeles, CA; 3 Department of Biomedical Physics, David Geffen School of Medicine, University of California Los Angeles, Los Angeles, CA; 4 Department of Bioengineering, Henry Samueli School of Engineering and Applied Science, University of California Los Angeles, Los Angeles, CA; 5 Center for Statistical Sciences, Brown University, Providence, RI; 6 Department of Diagnostic Imaging, Rhode Island Hospital and Alpert Medical School of Brown University, Providence, RI; 7 Radiology Associates of Clearwater, University of South Florida, Clearwater, FL; 8 Mallinckrodt Institute of Radiology, Washington University in St. Louis, St. Louis, MO, USA; 9 Neuro-Oncology Service, Tel Aviv Sourasky Medical Center, Israel; 10 Departments of Radiology and Neurosurgery, Henry Ford Hospital, Detroit, MI; 11 Department of Diagnostic Radiology, The University of Texas MD Anderson Cancer Center, Houston, TX; 12 A.A. Martinos Center for Biomedical Imaging, Massachusetts General Hospital, Harvard Medical School, Charlestown, MA; 13 Department of Neuro-Oncology, The University of Texas MD Anderson Cancer Center, Houston, TX; 14 Department of Radiology, Duke University Medical Center, Durham, NC, USA

**Keywords:** diffusion MRI, functional diffusion maps, functional diffusion mapping, glioblastoma, magnetic resonance imaging, bevacizumab

## Abstract

Functional diffusion mapping (fDM) is a cancer imaging technique that quantifies voxelwise changes in apparent diffusion coefficient (ADC). Previous studies have shown value of fDMs in bevacizumab therapy for recurrent glioblastoma multiforme (GBM). The aim of the present study was to implement explicit criteria for diffusion MRI quality control and independently evaluate fDM performance in a multicenter clinical trial (RTOG 0625/ACRIN 6677). A total of 123 patients were enrolled in the current multicenter trial and signed institutional review board-approved informed consent at their respective institutions. MRI was acquired prior to and 8 weeks following therapy. A 5-point QC scoring system was used to evaluate DWI quality. fDM performance was evaluated according to the correlation of these metrics with PFS and OS at the first follow-up time-point. Results showed ADC variability of 7.3% in NAWM and 10.5% in CSF. A total of 68% of patients had usable DWI data and 47% of patients had high quality DWI data when also excluding patients that progressed before the first follow-up. fDM performance was improved by using only the highest quality DWI. High pre-treatment contrast enhancing tumor volume was associated with shorter PFS and OS. A high volume fraction of increasing ADC after therapy was associated with shorter PFS, while a high volume fraction of decreasing ADC was associated with shorter OS. In summary, DWI in multicenter trials are currently of limited value due to image quality. Improvements in consistency of image quality in multicenter trials are necessary for further advancement of DWI biomarkers.

## Introduction

Approximately 20.6 people per 100,000 people in the United States are diagnosed with a primary brain tumor each year (1). GBM constitutes the most common and aggressive form of malignant glioma, occurring in ~54% of gliomas (1) or 3.2 per 100,000 US citizens, and carrying a dismal prognosis of a median survival of around 14 months (2) with <10% of patients surviving beyond 5 years after diagnosis. Currently, the standard of care for newly diagnosed GBM patients consists of maximum surgical resection, followed by radiotherapy plus concomitant and adjuvant temozolomide. At recurrence, however, very few therapeutic options exist. Currently, no treatment regimens have produced considerable therapeutic benefit in recurrent GBM (3).

Bevacizumab, a monoclonal antibody to VEGF (4) is now a common second-line treatment option for GBM patients that have failed the standard of care, particularly due to an apparent progression-free survival benefit shown in early clinical trials (5–7) compared with historic controls (2). These early results were based on a modified Macdonald criteria (8), which is limited in the evaluation of anti-angiogenic treatments due to the dramatic effect on vascular permeability resulting in decreased contrast enhancement (9,10). Diffusion-sensitive magnetic resonance imaging (MRI) biomarkers have shown some early promise as predictive tools (11) in bevacizumab therapy at recurrence. In particular, the functional diffusion map (fDM) technique, which evaluates voxel-wise changes in the apparent diffusion coefficient (ADC) over time, has shown utility as an early response biomarker in bevacizumab therapy in a single institution dataset consisting of uniform, high-quality diffusion MRI data (11). This technique, however, has not been evaluated in the context of a large multicenter trial with mixed quality of diffusion MRI data.

The aim of the present study was to implement explicit criteria for quality control and evaluate fDM performance using DWI data collected as part of RTOG-0625, a multicenter, randomized, phase II trial of bevacizumab with irinotecan or temozolomide in recurrent GBM.

## Materials and methods

The Radiation Therapy Oncology Group (RTOG), in collaboration with the American College of Radiology Imaging Network (ACRIN), both funded by the National Cancer Institute, conducted a prospective, randomized, phase II multi-center trial comparing bevacizumab with either irinotecan or temozolomide treatment in recurrent GBM (RTOG 0625/ACRIN 6677; ClinicalTrials.gov #NCT00433381; NCI-2009-00743). Twenty-four institutions both participated and had diffusion MRI data available for analysis, each obtaining institutional review board approval before subject accrual and conducting the trial with Health Insurance Portability and Accountability Act (HIPAA) compliance. Informed consent was obtained for all subjects.

### Study subjects

A total of 123 patients were enrolled in the current trial (Table I). All patients had recurrent histologically proven GBM or gliosarcoma with progression on MRI within 14 days after registration, ≥42 days after completion of radiation/temozolomide therapy, ≥28 days after surgical resection or cytotoxic therapy, as well as imaging or biopsy confirmation of true progressive disease rather than radiation necrosis after Gliadel placement or stereotactic radiosurgery. Detailed inclusion and exclusion criteria are available at http://www.acrin.org/Portals/0/Protocols/6677/RTOG%20062-ACRIN%206677.pdf (Section 3.0). Bevacizumab was administered to all patients (10 mg/kg intravenously, days 1 and 15 of a 28-day cycle). In the first arm, patients received temozolomide (75 mg/m^2^ per os, days 1–21 during the first 28-day cycle; 100 mg/m^2^ for cycle 2 and beyond in the absence of myelotoxicity). In the second arm, patients received irinotecan (125 mg/m^2^ intravenously, days 1 and 15 of a 28-day cycle). Standard of care MRI occurred at baseline, after every 2 cycles of treatment (every 8 weeks), and after completion or termination of treatment. Patients demonstrating benefit (stable or responding tumor) were treated for 12 cycles with optional extension to 24 cycles in the presence of continued benefit and absence of severe toxicity.

### Magnetic resonance imaging

Conventional MRI included pre-contrast T1-weighted, T2-weighted, T2-weighted FLAIR, and diffusion-weighted MRI (DWI). After intravenous injection of 0.1 mmol/kg of standard gadolinium-based contrast, an axial 2D spin-echo and 3D volumetric T1-weighted (T1+C) images were acquired. Patients participating in the optional advanced component of the trial had dynamic contrast-enhanced MRI, dynamic susceptibility contrast perfusion-weighted MRI, and/or MR spectroscopy at baseline, week 2 and after every 2 cycles of treatment.

Diffusion MR acquisition parameters varied widely across institutions despite specific ACRIN recommendations. Echo time (TE) varied from 64 to 111.9 ms (~200%), and by as much as 50% in the same patient during follow-up evaluations. Repetition time (TR) varied from 6 to 10 sec (~50%), *b*-values ranged from 0 and 700 to 0 and 1,200 sec/mm^2^, and in some cases diffusion tensor imaging (6–12 directions) was also acquired. In order to ensure relative consistency of ADC calculations across sites, measures of ADC were obtained from 2 *b*-values (typically a single *b*=0 sec/mm^2^ image and an image with higher diffusion weighting, or *b*=700–1200 sec/mm^2^. For DTI data, average trace images were used for this higher diffusion weighted image).

### Image registration

All images for each patient were registered to their own pre-treatment, post-contrast, 3D T1-weighted images with use of a mutual information algorithm and a 12-degree of freedom transformation using FSL (FMRIB; http://www.fmrib.ox.ac.uk/fsl/). This was followed by visual inspection to ensure adequate alignment. All images were interpolated to the resolution of baseline post-contrast T1-weighted images using trilinear interpolation. In cases with significant mass effect, attempts were made to align tumor regions exclusively. Regions of obvious misregistration (e.g. near ventricles or edge of the brain) were excluded from final fDM analysis.

### Quantitative quality control evaluation of diffusion MR data and image registration

Quality control (QC) evaluation was performed on both the diffusion MR data as well as the alignment between subsequent scans for use in fDM analysis. DWI at each scan date were evaluated in terms of the following factors: i) geometric distortion or artifacts on diffusion MR datasets; ii) ADC values within normal appearing white matter (NAWM) being within an acceptable range of ~0.4–1.0 μm^2^/ms; and iii) ADC values within cerebrospinal fluid (CSF) being within an acceptable range of ~2.5–4.0 μm^2^/ms. A 5-point quantitative scaling scheme was used for each of these factors as shown in Table II. The final QC score for each patient was calculated as the minimum QC value from each of the parameters in Table II. Additionally, if DWI data were not available for a particular patient, the QC score was zero.

### Region of interest (ROI) determination

In the present study, we chose to apply fDMs to regions of contrast-enhancing tumor on pre-treatment, post-contrast T1-weighted images. This approach has been shown to be the most predictive in other treatment settings (11,12). Additionally, this time-point likely contains the largest extent of contrast enhancing tumor for use in fDM evaluation, since bevacizumab therapy results in dramatic reduction of the volume of contrast enhancement in the majority of patients. We used a semi-automated process of: i) manually defining the relative region of tumor occurrence; ii) thresholding the post-contrast images using an empirical threshold combined with a region-growing algorithm; then iii) manually editing the resulting masks to exclude any obvious errors. For QC evaluations, a circular ROI (area, 1.5 cm^2^ or ~1.4 cm diameter) was placed in the contra-lateral NAWM and within the contra-lateral, anterior or posterior lateral ventricles for a measure of normal CSF.

### Functional diffusion map (fDM) calculation

After proper registration was visually verified, voxel-wise subtraction was performed between ADC maps acquired post-treatment and baseline, pre-treatment ADC maps. Individual voxels were stratified into three categories based on the change in ADC relative to the baseline ADC map. Red voxels represented areas where ADC increased beyond a ΔADC threshold of 0.4 μm^2^/ms, or ADC(+), and blue voxels represented areas where ADC decreased beyond a ΔADC threshold of 0.4 μm^2^/ms or ADC(−). These ΔADC thresholds (±0.40 μm^2^/ms) represent the 95% confidence interval for a mixture of normal appearing gray and white matter estimated from 69 patients with various tumor grades and follow-up time intervals ranging from 1 week to 1 year post-baseline (13). The fraction of ADC(+) and ADC(−) within the pre-treatment, post-contrast T1-weighted images [%ADC(+) and %ADC(−)] was subsequently used for fDM analysis.

### Independent radiological facility definition of disease progression

All local imaging was retrospectively transmitted to ACRIN for central review. Two primary readers and one adjudicator, each with neuroradiology Certificates of Added Qualification and 8, 6 and 3 years of post-fellowship experience, respectively, were trained via teleconference about 2D measurement techniques. Each primary reader was assigned 2 similarly trained core laboratory technologist and conducted independent image assessments. For each distinct contrast-enhancing target lesion as defined by Macdonald and RANO criteria (≥1 cm diameter, ≥1 cm from other enhancing lesions), the largest diameter of contrast enhancement and its maximum perpendicular diameter in the same plane were measured. 2D tumor area was computed by summing over all lesions the product of maximum perpendicular diameters. Each reader determined time of progression on 2D post-contrast T1-weighted images when there was >25% increase with respect to nadir in maximal cross-sectional enhancing areas or the appearance of any new enhancing tumor (9,14). Similarly, radiologic response was defined as ≥50% decrease with respect to baseline, confirmed on the subsequent time-point. Steroid dosage and clinical status were unavailable to ACRIN readers for the present study. The adjudicator settled discordant times to progression between primary readers by selecting the times to progression that were most correct in their opinion. The final measure of progression-free survival (PFS) for the present study was defined as the time from the first post-therapy scan used in fDM analysis until radiographic progression.

### Statistical analysis

A Kruskal-Wallis non-parametric test was used to compare ADC measurements in normal tissue across sites with 3 or more patients. Pooled variance two-sample t-tests were used to compare pre-treatment enhancing tumor volume, %ADC(+), or %ADC(−) between patients who progressed/expired vs. were progression-free at 6 months and those who expired at 12 months vs. those who were alive at 12 months from the first post-treatment MRI. Two-sample Satterthwaite t-tests were used if group variances were significantly different. A Cox-regression model was used to evaluate continuous measures of pre-treatment enhancing volume, %ADC(+) or %ADC(−) adjusted for age and gender, where the outcome was either PFS or overall survival (OS). Time-dependent receiver operating characteristic (ROC) analysis was performed for PFS or OS to determine the thresholds for %ADC(+) and %ADC(−) that maximized Youden’s index (sensitivity+specificity-1). The threshold values were used to divide %ADC(+) or %ADC(−) into two groups. Median PFS and OS as well as their curves within each group were estimated by the Kaplan-Meier method. Log-rank tests were conducted to compare the PFS (or OS) curves between the two groups of %ADC(+) [or %ADC(−)]. Data were examined separately for all usable DWI cases (QC ≥3) and cases with high quality DWI data (QC=5) to illustrate the effects of image quality on fDM analyses. P-values <0.05 were considered significant and P-values <0.1 were considered trending toward significance. All statistical data analyses were performed with SAS software, version 9.3 (SAS Institute Inc., Cary, NC, USA).

## Results

### Normal tissue ADC and quality control assessment

**Figure 1:**
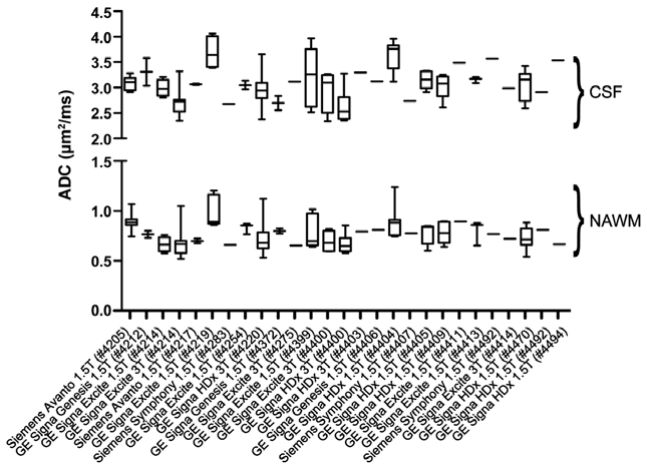
Mean ADC estimates for cerebrospinal fluid (CSF) and normal-appearing white matter (NAWM) across different sites, scanner manufacturers, and field strengths. Kruskal-Wallis non-parametric comparisons of CSF and NAWM in sites with 3 or more patients suggested ADC varied significantly by site (P<0.0001), with some sites showing systematically higher or lower ADC values in normal tissues.

The evaluation of pre-treatment ADC measurements within normal tissues for different sites, MR manufacturers, and acquisition techniques are shown in Fig. 1. In general, there was a wide variation in diffusion measurements within the various tissue types. The average coefficient of variance across all sites was 7.3% for NAWM and 10.5% for CSF. Kruskal-Wallis non-parametric comparisons of CSF and NAWM in sites with 3 or more patients suggested ADC varied significantly across sites (NAWM, P<0.001; CSF, P<0.001). Closer examination suggested that certain sites had systematically elevated or suppressed estimates of ADC within normal tissues.

**Figure 2:**
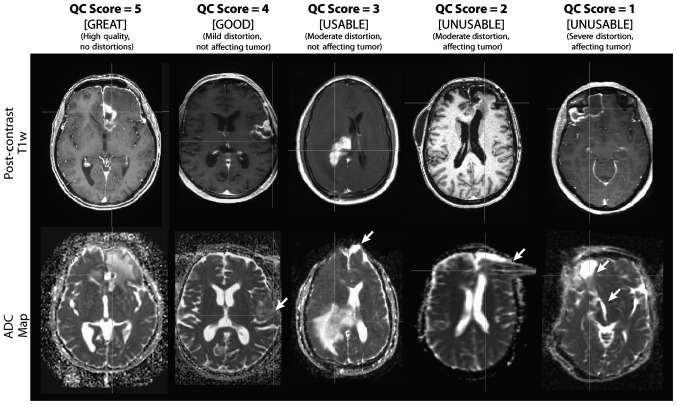
Example images for QC scores resulting from varying degrees of geometric distortion in ADC maps. QC score = 5 (great) reflects high-quality diffusion MRI data with no distortions. QC score = 4 (good) reflects mild geometric distortion that does not affect the tumor. QC score = 3 (usable) reflects moderate geometric distortion not affecting the tumor. QC score = 2 (unusable) involves images with moderate distortion that is affecting measurement of the tumor. QC score = 1 (unusable) involves severe distortion that is affecting measurement of the tumor.

Of the 123 patients with diffusion data available, 84 patients (68%) had adequate image quality (QC score ≥3) and 58 patients (47%) had high quality data (QC score =5). Fig. 2 shows example diffusion MR images from patients for various QC scores. The average QC score for all 123 patients was 3.37. Of the 84 patients with adequate diffusion MR information, ACRIN determined 3 cases ineligible for analysis, 3 cases were withdrawn due to no evaluable contrast-enhancing tumor, 2 cases were excluded due to no baseline MR scan after registration to 6677, and 12 patients progressed prior to the first imaging time-point, resulting in a total of 64 patients (52%) of total enrolled patients with evaluable data for fDM analysis (QC score ≥3) and a total of 46 patients (37%) of total enrolled patients with high quality fDM data (QC score=5).

### Study cohort and general fDM characteristics

Of the 64 patients with diffusion MR data available for fDM analysis (QC ≥3), 34 patients were male and the mean age for all patients was 57.3 years old ±11.2 SD. The average pre-treatment contrast enhancing volume was 18.5±16.9 cc SD, average %ADC(+) was 17.8±14.4% SD, and average %ADC(−) was 20.6±17.9% SD.

**Figure 3:**
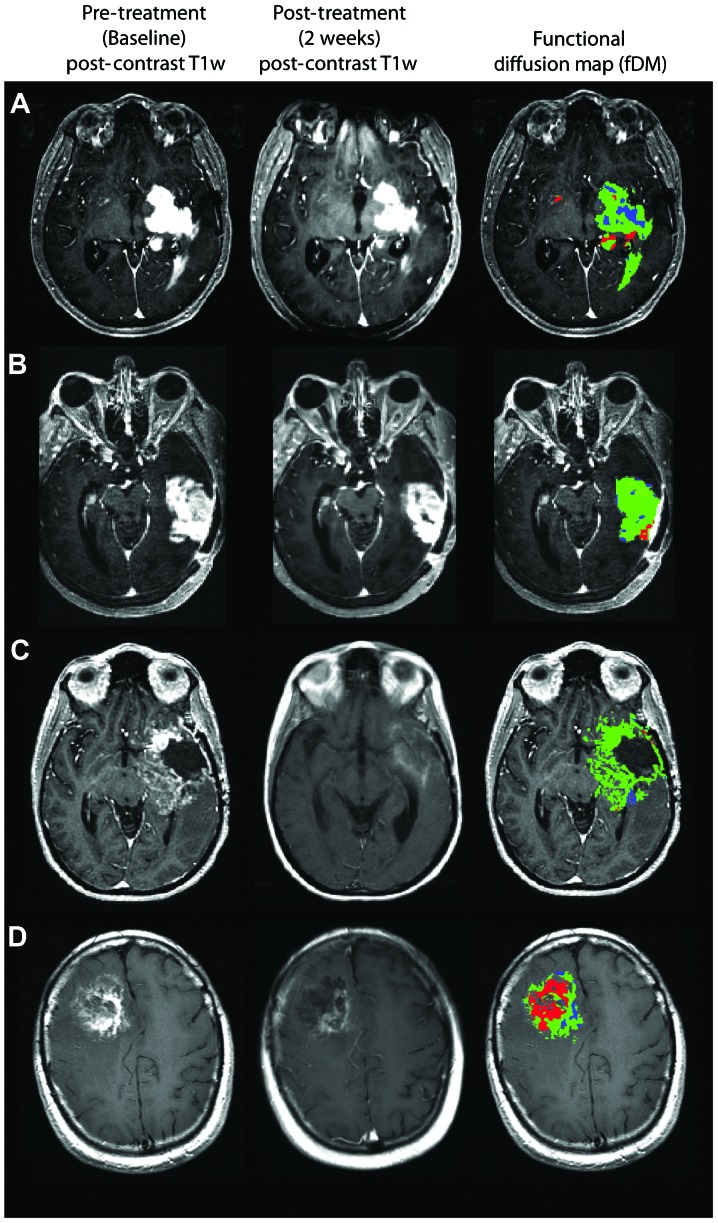
Examples of anatomical imaging and fDM response to bevacizumab and irinotecan or temozolomide. (A) This patient demonstrates a small change in enhancing tumor volume, but a relatively large proportion of the tumor with decreasing ADC (blue voxels). (B) This patient shows a similar change in enhancing tumor to the patient in (A), but shows very little change in ADC. (C) A patient with a dramatic change in contrast enhancement following therapy that is not accompanied by a substantial change in ADC. (D) A patient with a decrease in contrast enhancement that involves a large proportion of the tumor with increasing ADC (red voxels). Red voxels = ΔADC >+0.4 μm^2^/ms; blue voxels = ΔADC <−0.4 μm^2^/ms; green voxels = −0.4 μm^2^/ms ≤ΔADC ≤+0.4 μm^2^/ms.

Fig. 3 illustrates various examples of fDM response to therapy, which in many cases appeared independent of changes in anatomical images. For example, the patient in Fig. 3A showed little change in contrast enhancement after therapy, suggestive of stable disease or little response to therapy. fDM results in this patient showed a relatively large proportion of tumor with decreasing ADC (blue voxels), possibly suggestive of growing tumor or increasing cell density. Conversely, the patient shown in Fig. 3B demonstrated a similar change in anatomical imaging response, but little change on fDMs. Some patients showed a dramatic decrease in contrast enhancement following therapy and little change in ADC, such as the patient shown in Fig. 3C. Other patients showed a decrease in contrast enhancement that was accompanied by an increase in ADC (red voxels) similar to the patient shown in Fig. 3D.

### Progression-free survival (PFS)

#### Patients with DWI QC ≥3

**Figure 4:**
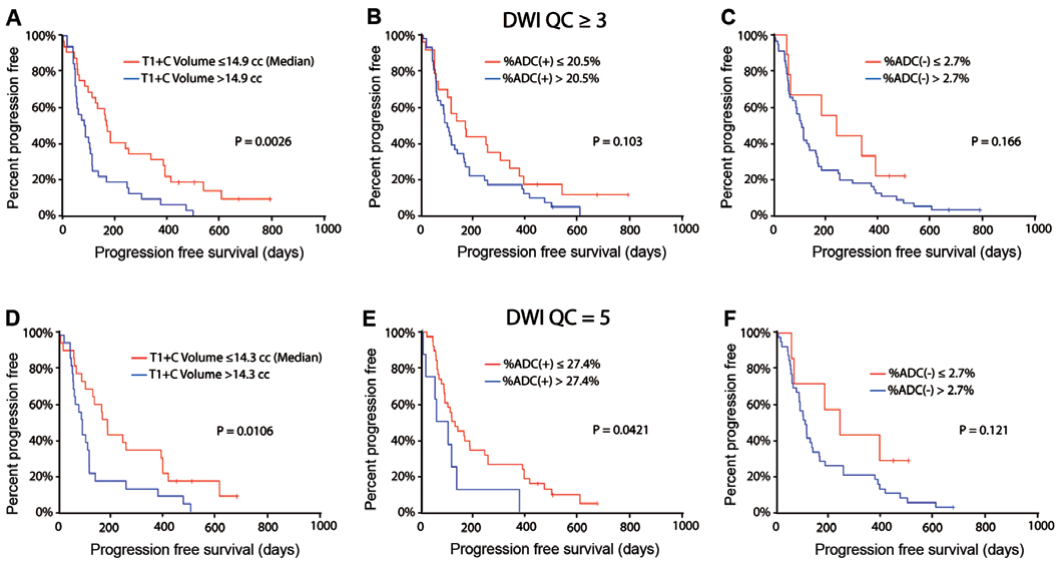
Pre-treatment contrast enhancing tumor volume and fDM response correlation of progression-free survival (PFS) for usable (QC ≥3) and high quality (QC=5) DWI data. (A) Stratification of PFS based on pre-treatment contrast enhancing volume (T1+C) in patients with usable DWI data (log-rank, P=0.0026). (B) Stratification of PFS based on the volume fraction of enhancing tumor with an increase in ADC [%ADC(+)] in patients with usable DWI data (log-rank, P=0.103). (C) Stratification of PFS based on the volume fraction of enhancing tumor with a decrease in ADC [%ADC(−)] in patients with usable DWI data (log-rank, P=0.166). (D) Stratification of PFS based on T1+C in patients with high quality DWI data (log-rank, P=0.0106). (E) Stratification of PFS based on %ADC(+) evaluated in patients with high quality DWI data (log-rank, P=0.0421). (F) Stratification of PFS based on %ADC(−) evaluated in patients with high quality DWI data (log-rank, P=0.121).

A total of 60 of 64 patients either progressed or expired at the time of final evaluation, while 43 of 64 patients either progressed or expired at 6 months from the first post-treatment time-point. Patients who were progression-free at 6 months showed no significant differences in pre-treatment volume of contrast enhancement and fDM characteristics from those who progressed or expired before 6 months (P>0.05). Continuous measures of enhancing volume were not significantly correlated with PFS (Cox regression: age, P=0.153; gender, P=0.214; pre-treatment enhancing volume, P=0.130); however, stratification of patients by median pre-treatment volume of contrast enhancement (14.9 cc) did show significant stratification of PFS (Fig. 4A; log-rank, P=0.003). Continuous measures of %ADC(+) and %ADC(−) from fDM analysis were not significantly correlated with PFS when adjusted for age and gender (Cox regression; P>0.05 for both %ADC(+) and %ADC(−)]. Youden’s index suggested an optimal cutpoint of %ADC(+) of 20.5% and %ADC(−) of 2.7% for PFS. Using these thresholds, patients with a large volume fraction of pre-treatment enhancing tumor with increasing ADC, or %ADC(+) >20.5 cc, had slightly worse PFS (median PFS = 167 vs. 98 days); however, this was not statistically significant (Fig. 4B; log-rank, P=0.103). Results also suggest patients with a large volume fraction of pre-treatment enhancing tumor with decreasing ADC at follow-up, or %ADC(−) >2.7, had a slightly shorter PFS (median PFS = 107 vs. 240 days), but this was also not statistically significant (Fig. 4C; log-rank, P=0.116).

#### Patients with DWI QC=5

For patients with high quality DWI data, a significant difference in pre-treatment contrast enhancing volume was observed between patients who were progression-free at 6 months and those who expired or progressed before 6 months (11.6 vs. 19.9 cc, P=0.027), but no significant differences were found in fDM characteristics between these patients (P>0.05). Continuous measures of pre-treatment contrast-enhancing tumor volume were significantly correlated with PFS (Cox regression: age, P=0.196; gender, P=0.810; pre-treatment enhancing volume, P=0.012). Consistent with these trends, stratification of patients by median pre-treatment volume of contrast enhancement (14.3 cc) demonstrated significant stratification of PFS (Fig. 4D; log-rank, P=0.011). Continuous measures of %ADC(+) and %ADC(−) from fDM analysis were not significant predictors for PFS when accounting for age and gender [Cox regression: P>0.05 for both %ADC(+) and %ADC(−)]. Youden’s index suggested a threshold of %ADC(+) of 27.4% and %ADC(−) of 2.7% for PFS in patients with high quality DWI. Results suggest patients with a large volume fraction of pre-treatment enhancing tumor with increasing ADC or %ADC(+) >27.4%, had significantly shorter PFS (Fig. 4E; median PFS =77 vs. 120 days; log-rank, P=0.042). Results also suggest patients with a large volume fraction of pre-treatment enhancing tumor with decreasing ADC at follow-up or %ADC(−) >2.7%, had a slightly shorter PFS (median PFS = 107 vs. 240 days), but this was not statistically significant (Fig. 4F; log-rank, P=0.121).

#### Overall survival (OS)

A total of 56 of 64 patients with evaluable DWI expired by the end of the study, while 45 of 64 patients expired by 12 months from the first post-treatment time-point. No difference in mean pre-treatment contrast enhancing volume or fDM characteristics were observed between patients alive at 12 months compared with those who expired at 12 months (P>0.05 for all metrics).

#### Patients with DWI QC ≥3

**Figure 5:**
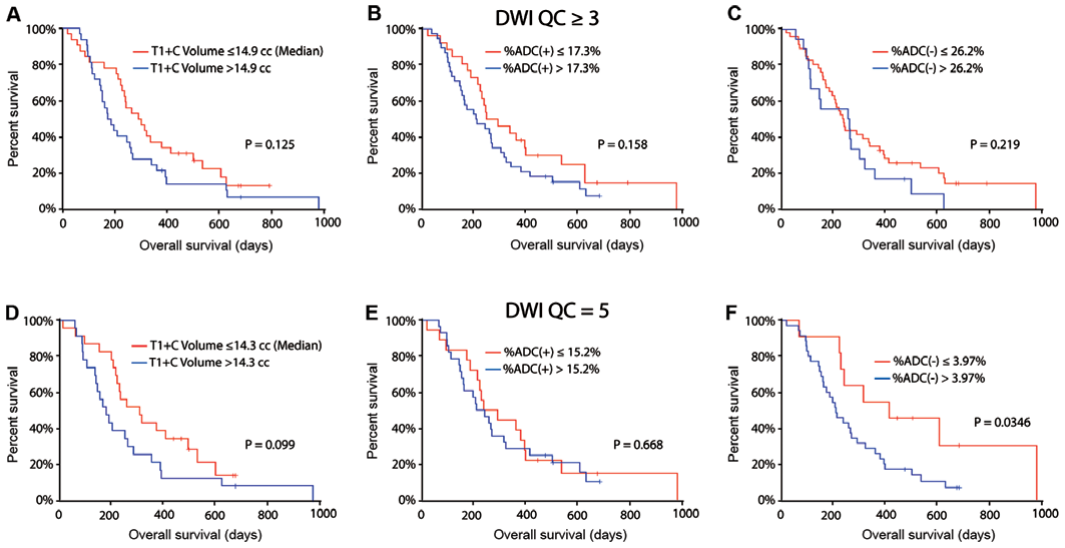
Pre-treatment contrast enhancing tumor volume and fDM response correlation with overall survival (OS) for usable (QC ≥3) and high quality (QC=5) DWI data. (A) Stratification of OS based on pre-treatment contrast enhancing volume (T1+C) in patients with usable DWI data (log-rank, P=0.125). (B) Stratification of OS based on volume fraction of enhancing tumor with an increase in ADC [%ADC(+)] in patients with usable DWI data (log-rank, P=0.158). (C) Stratification of OS based on volume fraction of enhancing tumor with a decrease in ADC [%ADC(−)] in patients with usable DWI data (log-rank, P=0.219). (D) Stratification of OS based on T1+C evaluated for patients with high quality DWI data (log-rank, P=0.099). (E) Stratification of OS based on %ADC(+) in patients with high quality DWI data (log-rank, P=0.668). (F) Stratification of OS based on %ADC(−) evaluated in patients with high quality DWI data (log-rank, P=0.0346).

Neither continuous measures of enhancing volume or fDM characteristics were significant predictors for OS [Cox, P>0.05 for volume, %ADC(+) and %ADC(−)]. When patients were stratified by median pre-treatment contrast-enhancing tumor volume (14.9 cc), no significant difference in OS was observed (Fig. 5A; log-rank, P=0.125). Th optimal cutpoints for %ADC(+) and %ADC(−) were 17.3 and 26.2% when the outcome was OS; however, neither %ADC(+) (Fig. 5B; log-rank, P=0.158) nor %ADC(−) (Fig. 5C; log-rank, P=0.219) significantly separated these groups in terms of OS.

#### Patients with DWI QC=5

For patients with high quality DWI data available, continuous measures of pre-treatment contrast-enhancing tumor was significantly correlated with OS (Cox, P=0.006 for volume, P=0.080 for age and 0.575 for gender). When patients were stratified by median pre-treatment enhancing volume (14.3 cc), a trend toward a difference in OS was observed (Fig. 5D; log-rank, P=0.099). Continuous measures of %ADC(+) and %ADC(−) were not significantly associated with OS (Cox, P>0.05 for fDM metrics). The optimal cutpoints for %ADC(+) and %ADC(−) in patients with high quality DWI data were 15.2 and 3.97%, respectively. The Kaplan-Meier curves between the two groups of %ADC(+) were not significantly different (Fig. 5E; log-rank, P=0.668). On the other hand, patients with a large volume fraction of pre-treatment enhancing tumor with decreasing ADC at follow-up, or %ADC(−) >3.97%, had a significantly shorter OS (Fig. 5F; median OS = 210 vs. 413 days; log-rank, P=0.035).

## Discussion

To the best of our knowledge, this is one of the first studies to define and implement specific diffusion MRI quality control criteria in the setting of a multicenter clinical trial in brain cancer. Results from the present study showed ~7.3–10.5% coefficient of variance in measurement of ADC across various sites. These results appear consistent with the measurements obtained by Chenevert *et al* (15), who estimated the variability of ADC in an ideal setting of an ice water phantom at ~5% when evaluated across vendors and platforms. It is important to note, however, that measures of ADC within a water phantom is monoexponential, thus, measurements of ADC may be quite resilient to the number of *b*-values and maximum *b*-value chosen, which may not be the case with normal neural tissues. More importantly, only 84 of the original 123 (68%) patients had usable DWI data free of distortion around the areas of tumor and only 58 of the original 123 (47%) patients had high quality DWI data with no distortions or ADC abnormalities. [In the end, only 64 patients (52%) had usable DWI data and 46 patients (37%) had high quality DWI data after patients were excluded based on other factors]. This degree of unusable data is particularly discouraging if diffusion MRI is to be considered a secondary response biomarker or a potential imaging endpoint in future prospective multicenter clinical trials.

The present study clearly demonstrates the importance of performing semi-quantitative QC in the context of advanced imaging in multicenter clinical trials. Functional diffusion mapping using high quality diffusion MRI acquired before and after administration of bevacizumab is a valuable imaging biomarker for predicting survival in recurrent glioblastoma patients treated with bevacizumab. Almost all fDM metrics showed improved stratification of short- and long-term PFS and OS when examining the highest quality DWI data (QC=5) compared with usable DWI data (QC ≥3). In particular, examination of high quality DWI data showed significant stratification of short- and long-term PFS when examining the volume fraction of pre-treatment enhancing tumor with increasing ADC [%ADC(+)], while the volume fraction of enhancing tumor with decreasing ADC [%ADC(−)] showed significant stratification of short- and long-term OS. When examining only the usable DWI data (QC ≥3), these trends were not statistically significant.

Although only a subset of data was evaluable in the present multicenter study, fDM results appeared to show some trends that were consistent and other trends that were inconsistent with previous studies. For example, previous fDM studies involving radiochemotherapy (12,16,17) in newly diagnosed malignant gliomas and bevacizumab (11) in recurrent GBM showed that patients with a low volume fraction of tumor with decreasing ADC [%ADC(−)] were more likely to have a longer PFS and OS. In the present study, we observed the same trend, however, results only showed statistical significance when examining %ADC(−) in terms of OS the subset of patients with high quality DWI data. Contrary to previous fDM reports, patients exhibiting a large volume fraction of enhancing tumor demonstrating an increase in ADC at first follow-up [%ADC(+)] appeared more likely to progress earlier than patients with a small volume fraction. Since all these patients were treated with bevacizumab, which tends to rapidly reduce the amount of vasogenic edema, it is conceivable that tumors demonstrating an increase in ADC following bevacizumab may represent those tumors to which vascular permeability has increased, indicating ineffective anti-angiogenic therapy.

It is important to point out that pre-treatment contrast enhancing tumor volume was one of the strongest correlates of survival in recurrent GBM patients treated with bevacizumab and chemotherapy. Results from the present study suggest that continuous measures of pre-treatment enhancing tumor were significantly correlated with PFS and OS when accounting for clinical covariates, particularly when examining patients with the highest quality MR data. This observation is consistent with a recent study (18) examining contrast enhancing tumor before and after bevacizumab treatment in a similarly structured phase II multicenter study in recurrent GBM patients treated with bevacizumab monotherapy or bevcizumab and irinotecan. As measures of contrast enhancing tumor remain the gold standard for response assessment and estimating tumor burden in malignant gliomas, it is important to compare emerging imaging biomarkers with this standard to determine if they truly add clinical benefit.

A number of limitations and possible explanations for the relatively poor fDM performance should be addressed. First, the present study involved calculation of ADC given only 2 *b*-values, while the National Cancer Institute recommends that at least 3 *b*-values be acquired (0, >100 and >500 sec/mm^2^) for estimation of perfusion-insensitive ADC (19). Additionally, many sites did not comply with the recommended diffusion MRI protocols, nor was there a mechanism in place for real-time feedback of image quality as diffusion MRI was considered a secondary measurement to standard anatomic imaging techniques. Another potential limitation was the potential influence of geometric distortions on ADC measurements. Woodworth *et al* (20) recently showed that post hoc non-linear distortion correction of diffusion MR images to high-resolution T2-weighted images can improve diffusion measurements in brain tumors, demonstrating that subtle distortions can cause significant differences in ADC measurements. A similar approach could have been used in the present study to improve ADC measurements, even in patients with usable data (QC ≥3). Similarly, the use of a rigid-body image registration algorithm to align serial ADC maps to baseline ADC maps poses another potential limitation. Significant changes in mass effect from tumor growth or shrinkage, or intracranial pressure changes induced by changes in the extent of vasogenic edema may cause inaccuracies in the alignment between the diffusion MR datasets. A recent study by Ellingson *et al* (21) showed improved fDM performance in the context of bevacizumab therapy by using non-linear registration of ADC maps over time. It is conceivable that a similar approach may also have improved fDM performance in the context of the current study, which also involved similar therapies and registration challenges.

In conclusion, the present study suggests diffusion MRI data collected as part of a multicenter trial for brain tumors may be of limited value, due particularly to the wide variety in image quality across sites, vendors and acquisition protocols. In data deemed usable, fDM results showed similar trends but lower correlations compared with previous single-institution trials involving relatively high-quality diffusion data with homogeneous acquisition protocols. Stratification of survival using fDM metrics were substantially improved by examining a subset of patients with high quality DWI data, suggesting image quality may have a significant impact on fDM performance. Future studies aimed at improving the consistency of image quality in multicenter trials are necessary for further advancement of diffusion MR biomarkers.

## Figures and Tables

**Table I:** Summary of sites and number of patients enrolled.

Site	No. of patients
4205 - Barnes Jewish Hospital	7
4212 - Thomas Jefferson	2
4214 - MD Anderson	19
4217 - University of Iowa	2
4219 - Sloan Kettering	6
4220 - University of Rochester	1
4254 - Medical College of Wisconsin	3
4275 - Henry Ford	22
4283 – Akron General Medical Center	2
4372 - St. John’s Health System	1
4399 - St. Luke’s	5
4400 - Tel-Aviv Medical Center	13
4403 - Mt. Diablo	1
4404 - JFK	1
4405 - LDS	7
4406 - Arizona Oncology Serv @ SJHMC	1
4407 - Virginia Mason Medical Center	5
4409 - Carolina’s Medical Center/Levine Cancer Ctr	6
4411 - N. Rockies Regional Cancer Center	1
4413 - Anne Arundel Medical Center	3
4414 - Alta Bates Comprehensive Cancer Center	1
4470 - Yale University	1
4492 - University of Chicago	12
4494 - UCLA	1
Total	123

**Table II:** Quantitative quality control definitions for diffusion MRI and fDM analysis.

Parameter	Score = 1 (Unusable)	Score = 2 (Unusable)	Score = 3 (Usable)	Score = 4 (Good)	Score = 5 (Great)
Distortion/artifacts	Severe, affecting tumor	Moderate, affecting tumor	Moderate, not affecting tumor	Mild, not affecting tumor	No distortion or artifacts
ADC values (NAWM)	Negative values	Non-physiological range (0–0.4 μm^2^/ms)	Lower or higher than normal, but within physiological range (e.g. 0.4–0.6 μm^2^/ms; 0.8–1.0 μm^2^/ms)		Within normal range (0.6–0.8 μm^2^/ms)
ADC values (CSF)	Negative values	Non-physiological range (0–1.5 μm^2^/ms; 4.0+ μm^2^/ms)	Lower or higher than normal, but within physiological range		Within normal range for CSF
Registration of ADC maps with Baseline ADC maps	Severe misalignment, tumor not aligned	Moderately misaligned, tumor not aligned	Moderately misaligned, but tumor is aligned	Slightly misaligned, but tumor is largely aligned	Perfectly aligned
